# BEHIND THE WALLS: THE IMPLICATIONS OF ASSISTED LIVING BUILDING CODES ON RESIDENT SAFETY

**DOI:** 10.1093/geroni/igae098.3809

**Published:** 2024-12-31

**Authors:** Adam Pennavaria, Lindsey Smith, Cassandra Hua, Paula Carder, Eric Jutkowitz, Kali Thomas

**Affiliations:** Oregon Health and Science University, Portland, Oregon, United States; Oregon Health & Science University, Portland, Oregon, United States; University of Massachusetts Lowell, Lowell, Massachusetts, United States; OHSU-PSU School of Public Health, Portland, Oregon, United States; Brown University, Brown University, Rhode Island, United States; Johns Hopkins University, Baltimore, Maryland, United States

## Abstract

As the US population collectively ages, issues related to assisted living (AL) resident safety have become an increasingly pressing issue for the general public, reflected by major media reporting in the last year. The building codes that govern ALs play an important role in mitigating the risks associated with falls, wandering, and other hazards, especially for residents living with Alzheimer’s disease and related dementias. We sought to understand how states vary in their approaches to building code regulations that might impact resident safety. We used search term-based text retrieval to identify areas of text relevant to locks, alarms, and egress restriction. A preliminary analysis of 2023 AL policy text by state suggests that a majority of states’ AL policy regulations contain some kind of reference to requirements for locks, alarms, or other type of egress restriction, with a few outliers – regulations in Nebraska, Minnesota, South Carolina, and Tennessee appear to make no mention of any of these subjects, while New Mexico, Texas, Washington, and others include multiple references to each. For instance in NM, residents must have the ability to safely leave the building, so memory care units rely on alarms and provide access to secure outdoor environments. 38 states’ regulations (75%) refer to locks, 40 states (78%) refer to alarms, and 27 states (53%) refer to limiting/restricting egress. This research has implications for understanding the relationships between building code requirements and resident safety. It provides insight for those who seek to improve safety standards in AL communities.

